# Double‐balloon endoscopy for duodenal diverticulitis with calculus after intestinal reconstruction: A case report

**DOI:** 10.1002/deo2.70044

**Published:** 2024-12-15

**Authors:** Kai Toyoshima, Yunosuke Takishin, Kiko Toda, Katsuma Nakajima, Yutaro Otuka, Daisuke Yokoyama, Tetsuhito Muranaka, Yasuyuki Kunieda

**Affiliations:** ^1^ Department of Internal Medicine Wakkanai City Hospital Hokkaido Japan

**Keywords:** case report, double‐balloon endoscopy, duodenal diverticulitis, duodenal fistula, small intestine

## Abstract

Small‐bowel diverticulosis is relatively common, but there is no set treatment strategy for duodenal diverticulitis with stone impaction. A woman aged in her 70s presented with a chief complaint of abdominal pain, and she had been reconstructed by the Roux‐en‐Y method after total gastrectomy. We performed an enhanced computed tomography which revealed edematous wall thickening of the duodenum. We diagnosed her with duodenal diverticulitis and treated them with antibiotics but her disease was not cured, we therefore attempted endoscopic stone removal as a nonoperative treatment. After stone removal with a nonoperative procedure, when we contrasted the duodenal papillary diverticulum, it was found to form a fistula on the other side, and the successful treatment made her discharged on the 17th day. The duodenal diverticulitis with calculus is extremely rare, and there is no report to treat it using double‐balloon endoscopy, therefore we report this case with a literature review.

## INTRODUCTION

Small‐bowel diverticulosis is relatively common and can be discovered incidentally during celiotomy, endoscopy, or radiographic imaging studies. However, there is no set treatment strategy for duodenal diverticulitis with stone impaction. Although it was reported that the low incidence of complications associated with duodenal diverticula justifies a nonoperative approach,^1^ it is technically difficult to approach the duodenum to the Roux‐en‐Y reconstructed intestinal tract after total gastrectomy. We report a rare case of diverticulitis due to stone impaction in a duodenal diverticulum after total gastrectomy. Written informed consent was obtained from the patient for publication of this case report and accompanying images.

## CASE REPORT

**FIGURE 1:**
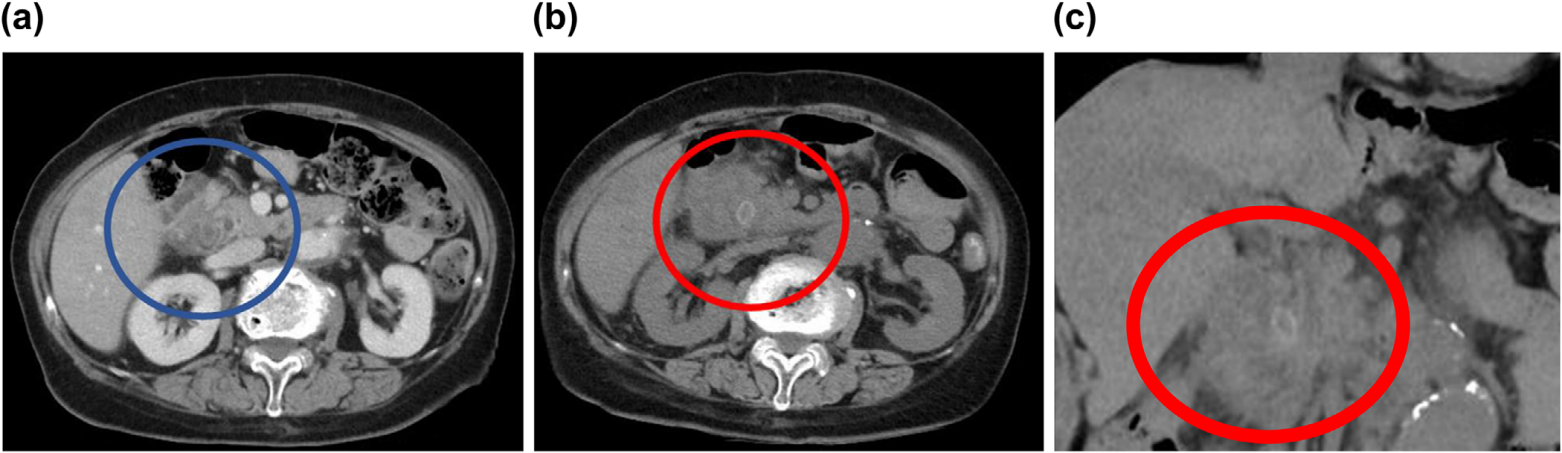
(a) Contrast‐enhanced computed tomography (CT) scan on admission revealed edematous wall thickening of the duodenum. (b) A re‐examination of plain CT images revealed the presence of calcified duodenal diverticular calculus, leading to a diagnosis of duodenal diverticulitis with parapapillary calculus. (c) The plain CT coronal section showed the calcified duodenal diverticular calculus as a diverticulum located beside the duodenal papillae, slightly toward the mouth on the side of the pancreatic head.

**FIGURE 2:**
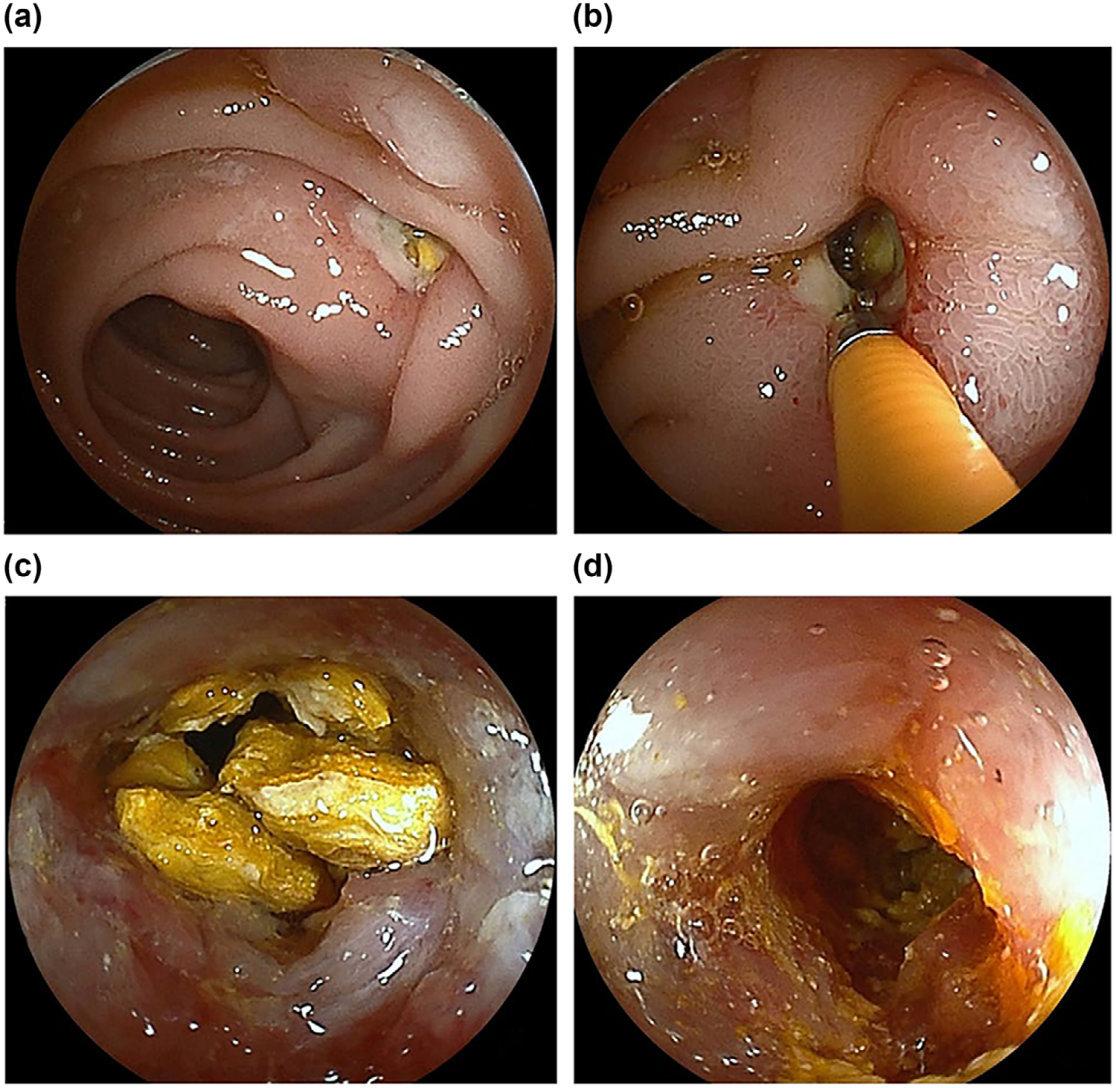
When the double‐balloon endoscope first reached a certain papillary diverticulum in the duodenum, a small stone was visible in the duodenal diverticulum, from which pus was draining (a). We used forceps to break up the stone in the diverticulum (b), drain it (c), and flush the diverticular cavity with water (d).

**FIGURE 3:**
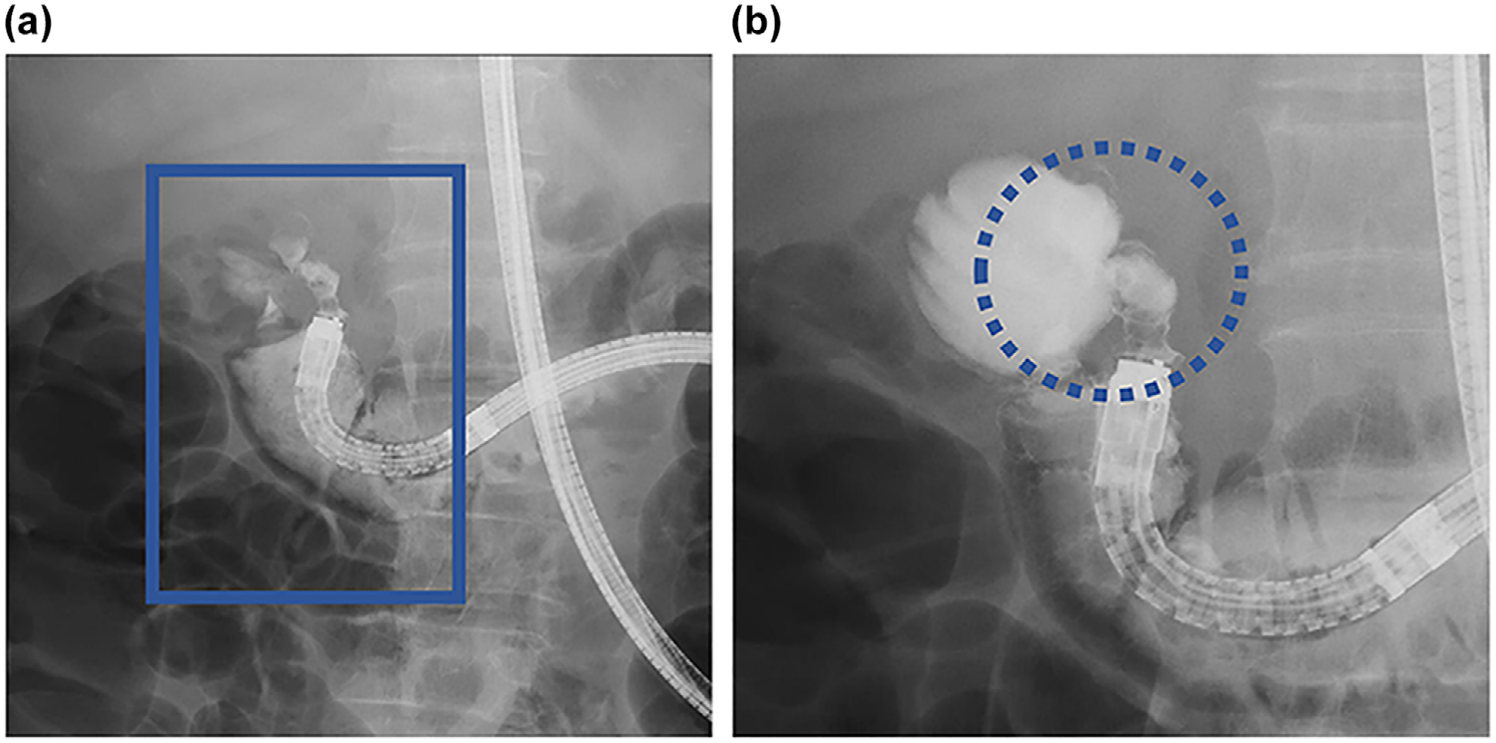
We contrasted the diverticulum and found that a fistula had formed from within the diverticulum into the lumen of the blind end of the afferent loop.

The patient, a woman in her 70s, presented with a chief complaint of abdominal pain. She developed a sudden onset of epigastric pain during the night and presented to our emergency department. One of her medical histories was gastric cancer, for which a total abdominal gastrectomy had been performed and Roux‐en‐Y reconstruction was performed. She was admitted to the hospital as an emergency and we started treatment with antibiotics. On admission, her abdomen was flat and soft with localized tenderness in the pericardial area, her consciousness was clear, her temperature was 35.8°C, her pulse was 72 bpm and her blood pressure was 148/74 mmHg. On admission, blood tests showed an elevated white blood cell count of 20,700/mm^3^ with a predominance of neutrophils and an elevated C‐reactive protein of 6.38 mg/dL (Table 1). There were no elevated hepatobiliary enzymes. Contrast‐enhanced computed tomography (CT) scan on admission showed edematous wall thickening of the duodenum (Figure 1a). Treatment was started with cefmetazole, but the patient continued to have a fever, and the inflammatory response did not improve, so the plane CT scan was repeated. It revealed a duodenal diverticulum stone and an increased concentration of surrounding fatty tissue, which allowed us to diagnose duodenal diverticulitis associated with a parapapillary stone (Figure 1b,c) which could have been identified by the high CT value at the first time enhanced CT. We therefore attempted endoscopic stone removal as a nonoperative treatment on day 9. As she had undergone a total gastrectomy and Roux‐en‐Y reconstruction, we advanced to the import leg using a small‐bowel double‐balloon endoscope and found a diverticulum. On observation, we found a stone lodged in the duodenal diverticulum and a white purulent discharge from it. The diverticulum was located beside the duodenal papillae, slightly toward the mouth on the side of the pancreatic head. We used clamping forceps (Radial Jaw 4 Standard Capacity; Boston Scientific) to crush the stone in the duodenal diverticulum, which was subsequently washed out with water via the scope channel, gradually widening the entrance to the diverticulum (Figure 2). Finally, we could remove the stone. After that, we used Meglumine sodium amidotrizoate to contrast the diverticulum and found that a fistula had formed from within the diverticulum into the lumen of the blind end of the afferent loop (Figure 3). The procedure was terminated without suture as there was no leakage of contrast material out of the bowel. Her subsequent clinical course was good, with rapid improvement in fever and inflammation, and she was discharged on the 17th day.

**TABLE 1:** Laboratory data on admission.

WBC	20,700	/µL	TP	6.8	g/dL
Neut	92.4	%	T.Bil	0.7	mg/dL
Lymp	6.1	%	AST	28	IU/L
RBC	207	×10^4^/µL	ALT	15	IU/L
Hb	7.9	g/dL	LDH	299	IU/L
Plt	24.8	×10^4^/µL	γ‐GT	17	IU/L
			ALP	269	IU/L
PT	12.9	s	AMY	76	IU/L
APTT	27.9	s	BUN	19.3	mg/dL
D‐dimer	3.07	µg/mL	Cre	0.70	mg/dL
			Na	138	mEq/L
CEA	3.0	ng/mL	K	3.2	mEq/L
CA‐19‐9	7.2	U/mL	CRP	6.38	mg/dL

## DISCUSSION

This is the first report in the world to treat duodenal diverticulitis with calculus using a double‐balloon endoscope in a bowel reconstructed after Roux‐en‐Y total gastrectomy. Duodenal diverticula can cause combined pancreaticobiliary obstruction through multiple mechanisms such as stasis‐induced primary choledocholithiasis, stasis‐induced intradiverticular enterolith, or longstanding diverticulitis, causing stenosing fibrosing papillitis or a combination of more than one of these mechanisms.^2^ Tsukamoto et al. reported two cases of perforated duodenal diverticulum and recommended that although surgical intervention is the standard treatment of duodenal diverticula, conservative therapy is also an option.^3^ Gurala et al. reported that among small intestinal diverticula, duodenal diverticula are more frequent, followed by diverticula of the jejunum and ileum. A jejunal diverticulum is usually asymptomatic; sometimes patients complain of vague chronic symptoms like malabsorption, pain, or nausea that easily lead to misdiagnosis. Complications are rarely reported, only in 10% of patients.^4^

In this case, the patient survived because the fistula formation was in the intestinal tract, but cases of duodeno‐sigmoid, duodeno‐retroperitoneal, or duodenal‐vena cava fistula formation have also been reported^5^, ^6^, ^7^, ^8^ and curative surgery such as bypass, colectomy, or revascularization surgery, should be considered postdiagnosis. However, there are no coherent reports on whether to perform these invasive procedures due to the risk of complications from the procedure. Based on the present case, it is worth considering whether the procedure, if performed, can be treated endoscopically first, rather than considering surgery first. If the bile duct stones or gallbladder stones were present, the possibility of biliary‐diverticulum fistula being the cause of the stones in the diverticulum should be investigated. Herein, CT images revealed no stones in the bile duct or gallbladder.

Recurrence of duodenal diverticular stones has never been reported; however, if the stones form because of the presence of diverticula, future recurrence is possible. The present report provides encouraging evidence for such recurrence being treated endoscopically.

Most cases of diverticulitis of the colon can be resolved with conservative treatment. However, the present case involved duodenal diverticulitis with stones, which was refractory to conservative treatment for 9 days. This was successfully treated with endoscopic removal of the stone.

Short double‐balloon endoscopy is feasible for the management of biliary disease in patients undergoing Roux‐en‐Y gastrectomy or hepatectomy^9^ and double‐balloon ERCP is well tolerated, safe, and has a high success rate in patients with Roux‐en‐Y reconstructed bowel.^10^ However, we are the only authors to report on the nonoperative treatment of duodenal diverticulitis with stones using a double‐balloon endoscope.

In the present case, we report on the successful nonoperative treatment of duodenal diverticulitis with stones using a double‐balloon endoscope.

## CONFLICT OF INTEREST STATEMENT

None.

## ETHICS STATEMENT

The authors report the details of the patient's case in accordance with the ethical standards of the Declaration of Helsinki.

## PATIENT CONSENT STATEMENT

Written informed consent was obtained from the patient for publication of this case report and accompanying images.

## CLINICAL TRIAL REGISTRATION

Not applicable.

## Data Availability

Not applicable.
